# Difference‐Makers for Robust Implementation of a Nursing Home Advance Care Planning Embedded Pragmatic Clinical Trial

**DOI:** 10.1111/jgs.70289

**Published:** 2026-01-30

**Authors:** Susan E. Hickman, Edward J. Miech, Timothy E. Stump, Wanzhu Tu, Kathleen T. Unroe

**Affiliations:** ^1^ School of Nursing Indiana University Indiana USA; ^2^ School of Medicine Indiana University Indiana USA; ^3^ IU Center for Aging Research Regenstrief Institute Inc. Indiana USA; ^4^ Department of Emergency Medicine Indiana University School of Medicine Indiana USA; ^5^ Department of Biostatistics and Health Data Science Indiana University Indiana USA

**Keywords:** coincidence analysis, dementia, palliative care

## Abstract

**Introduction:**

Embedded pragmatic clinical trials are an ideal way to develop and evaluate evidence‐based interventions in the nursing home (NH) environment to facilitate streamlining implementation after study completion. However, there is minimal information available about the necessary and sufficient conditions of “difference makers” for robust implementation of pragmatic interventions in the NH setting.

**Methods:**

The “Aligning Patient Preferences—a Role Offering Alzheimer's patients, Caregivers, and Healthcare Providers Education and Support” (APPROACHES) embedded pragmatic trial is designed to test and evaluate a staff‐led advance care planning (ACP) intervention for residents with dementia in 128 NHs (64 intervention, 64 control). Coincidence Analysis, a case‐based approach to data analysis that draws upon Boolean algebra and set theory, was applied to identify key difference‐makers for robust implementation. This analysis focused on the 44 intervention NHs that implemented at least one of two implementation processes: site visits and/or monthly calls.

**Results:**

Eighteen of 44 (41%) sites in the analysis robustly implemented the APPROACHES intervention as reflected by > 75% of residents having a documented ACP conversation. The Coincidence Analysis revealed two pathways directly linked with robust pragmatic implementation: (1) no executive director turnover during the observation period combined with site participation in monthly calls with peers; and (2) higher rates of baseline hospitalization (3.96–7.0 per 1000 resident‐days alive) combined with a low number of certified beds. In contrast, leadership instability as reflected by administrator turnover, high number of certified beds, and a lack of participation in monthly calls with peers was associated with poorer performance.

**Discussion:**

Findings from this study suggest that leadership stability and engagement with peers were essential drivers of robust implementation of the APPROACHES ACP Specialist intervention. Coincidence Analysis is a useful tool for understanding how implementation conditions are associated with robust implementation in embedded pragmatic clinical trials.

## Summary

- Key Points
  ○ Coincidence Analysis is a newer methodology that is ideally suited to explore implementation conditions associated with outcomes in embedded pragmatic clinical trials.
  ○ Implementation conditions associated with successful implementation of the ACP Specialist Program in nursing homes include leadership stability and engagement with peers.
  ○ Leadership instability as reflected by nursing home administrator turnover, a high number of certified beds representing a higher caseload, and a lack of participation in monthly calls with peers was associated with poorer implementation of the ACP Specialist Program.
- Why this study is important
  ○ Understanding the conditions needed to successfully implement programs in nursing homes like this systematic advance care planning program through novel methodologies like Coincidence Analysis is of importance to researchers designing studies in this setting as well as policymakers or health system leaders encouraging uptake of evidence‐based programs.

Nursing homes (NHs) are considered a challenging setting for clinical research and NH residents are under‐represented in clinical trials as a result [1, 2, 3]. A key issue in traditional explanatory trials is the need for a controlled experimental environment that is difficult to sustain in the NH setting. An alternative approach is the more flexible embedded pragmatic clinical trial (ePCT), which is designed to test interventions in real world settings with the goal of increasing the likelihood that successful interventions can be easily adopted and implemented [4, 5]. ePCTs require broad eligibility criteria, flexible intervention delivery by clinicians and staff with minimal training requirements, and the use of existing secondary data to assess outcomes rather than direct data collection. Pragmatic implementation of interventions can vary significantly based on the complexity of the intervention delivery due to both internal and external factors that impact implementation and intervention fidelity [6].

As a result of investments by the National Institutes of Health, there are increasing numbers of ePCTs, including significant growth in NH ePCTs due to a pressing need to grow the science of caring for persons living with Alzheimer's disease and related dementias [7, 8]. An early pioneer of this model in NHs was the PROVEN trial, designed to evaluate the effect of advance care planning (ACP) videos on hospitalizations in 119 intervention NHs in comparison to 241 control NHs. The intervention found no differences in hospital transfers per 1000 person‐days alive in intervention versus control groups. However, a major limitation of PROVEN was relatively low uptake of the intervention. NH engagement ranged from 0% to 40% and only 22% of residents with advanced illness viewed the ACP videos [9].

The APPROACHES (Aligning Patient Preferences—a Role Offering Alzheimer's patients, Caregivers, and Healthcare Providers Education and Support) Trial was a pragmatic trial of an ACP program implemented and evaluated in 128 NHs (64 intervention, 64 control) [10]. The model builds on the ACP strategy used in the Optimizing Patient Transfers, Impacting Medical quality, Improving Symptoms—Transforming Institutional Care (OPTIMISTIC) [11], a multicomponent intervention that successfully reduced potentially avoidable hospitalizations of long‐stay NH residents by nearly 40% in comparison to a matched control group [12].

We sought to identify the conditions that were necessary and sufficient for successful implementation in APPROACHES using Coincidence Analysis, an innovative mathematical, cross‐case approach applied in prior work that draws upon Boolean algebra, logic and set theory [13, 14, 15, 16, 17, 18, 19, 20, 21]. Coincidence Analysis offers a case‐based approach to data analysis that can be used to pinpoint what makes a difference, for whom, and under what conditions through its capacity to model conjuncts (when the joint appearance of several conditions produces the outcome) and disjuncts (when different pathways ultimately lead to the same outcome) [22]. Given that real‐world implementation processes and outcomes are often complex and context‐sensitive, Coincidence Analysis has recently gained traction in both health services research and implementation science, with over 50 articles published in the peer‐reviewed literature in these two fields alone since 2020 [23]. In this analysis, we applied Coincidence Analysis to identify the particular bundles of implementation conditions that consistently distinguished higher‐performing NHs from lower‐performing NHs (and vice versa) while taking into account context‐specific differences across cases. Accordingly, we considered both context and process conditions that could serve as potential “difference‐makers” that resulted in NHs having more ACP conversations with eligible NH residents, as well as conditions resulting in sites having *fewer* ACP conversations.

## 1 Methods

### 1.1 Setting

The APPROACHES intervention was implemented in 64 NHs located in eight states between September 1, 2021 and August 30, 2022 [10]. The study was reviewed and approved by the Indiana University Institutional Review Board and a waiver of informed consent was obtained.

### 1.2 Study Design

APPROACHES was delivered by specially trained, existing NH staff selected by the NH who were called ACP Specialists. ACP Specialists were expected to devote 20% effort to the role. In some NHs, more than one ACP Specialist was selected and responsibilities were shared. Each ACP Specialist was assigned an online training that included an initial 1‐hour overview and seven additional modules focused on foundational education about ACP, goals of care, and life‐sustaining treatments as well as an overview of the ACP Specialist's role. ACP Specialists were instructed to engage in 10 conversations per month and were provided with tools and resources to support this work including a worksheet to track progress on monthly assignments, an ACP facilitation guide, a toolkit of educational materials to share with residents, families, as well as other NH staff, and template letters for communicating with family caregivers. The outcome of each conversation was documented in an ACP documentation template that was embedded in the NH electronic health record (EHR). Support was also provided for corporate leads at the two partner NH companies to oversee implementation and meet monthly with the project team. The corporate leads were provided with implementation audit tools as well as the number of documented conversations by NH. They also were responsible for tracking training completion and turnover, leading monthly peer support calls for ACP Specialists to share implementation strategies, highlight successes, problem‐solve challenges with each other, as well as disseminate a monthly newsletter to ACP Specialists with case studies and frequently asked questions [10].

### 1.3 Participants

Any individual who was admitted to a participating NH during the study intervention period was eligible for inclusion. However, the ACP Specialists were instructed to prioritize conversations with persons living with dementia and their family caregivers.

### 1.4 Measures

We identified relevant and accessible secondary data related to implementation conditions and organized it using the Contextual Framework for Implementation Research [24, 25, 26]. The measures were constructed at the level of the NH to reflect factors potentially associated with implementation, as shown in Table 1. Consistent with the pragmatic design, there was no primary data collection.

**TABLE 1:** Advance care planning specialist program implementation condition candidate factor “Difference Makers,” Data Elements, and data sources used in the coincidence analysis.

Candidate factors	Data elements	Data sources
APPROACHES intervention characteristics	Prompt for conversation	Advance Care Planning (ACP) Documentation Template
	Average length of conversation	ACP Documentation Template
	Average # conversations per resident with documented advance care planning	ACP Documentation Template
	Outcome of ACP conversation	ACP Documentation Template
Inner setting—nursing facility characteristics	Baseline hospitalization rate	Minimum Data Set 3.0 data purchased from the Centers for Medicare and Medicaid Services
	Overall quality ratings	CMS Five‐Star Rating [27]
	Nurse practitioner in facility	Corporate Lead
	Facility size	CMS Provider Data
Outer setting—external environment	Rural/urban location	Rural/Urban Continuum Codes [28]
Individuals involved	Percent ethnic/racial minority	Long‐Term Care: Facts on Care in the US [29]
	ACP specialist turnover	Corporate Lead
	Number of ACP specialists	ACP Documentation Template
	Resident makes own decisions	ACP Documentation Template
	Primary decision‐maker for ACP	ACP Documentation Template
	Executive director turnover	Corporate Lead
	Staff turnover	Corporate Lead
Process of implementation	Facility received a site visit	Corporate Lead
	Participation in monthly phone calls	Corporate Lead

*Note*: The Contextual Framework for Implementation Research domains were used to organize the implementation condition candidate factors [25].

#### 1.4.1 APPROACHES Intervention Characteristics

Characteristics of the ACP Specialist intervention included: prompt for conversation (proactive or reactive); average length of conversation; average number of conversations per resident; and conversation outcomes (new orders vs. confirmed existing orders).

#### 1.4.2 Inner and Outer Characteristics

Inner characteristics included: baseline facility hospitalization rates divided into quartiles; overall quality star ratings [27]; presence of a nurse practitioner in the facility; and facility size as determined by the number of certified beds per facility divided into tertiles [30]. The only outer characteristic was facility location (i.e., rural vs. urban setting) determined using facility zip codes [28].

#### 1.4.3 Individuals Involved

Characteristics of the residents involved included: percent ethnic/racial minority residents in the NH [29]; proportion of residents who were able to make their own decisions during ACP conversations; and percent of conversations in which the primary decision‐maker was the surrogate. Staff characteristics included: Executive Director turnover; proportion of staff turnover; ACP Specialist turnover; and the number of ACP Specialists in the NH.

#### 1.4.4 Process of Implementation

Two implementation process measures were included: whether the facility received an in‐person site visit from the corporate lead and whether the ACP Specialists routinely participated in monthly calls with peers and the corporate lead.

### 1.5 Outcome

Our primary implementation outcome was the proportion of residents living with dementia who had been approached to engage in ACP conversations during the study intervention period. This number was calculated using the number of residents with documentation of an ACP encounter in the EHR divided by the total number of residents present at the facility for at least 1 day. Robust implementation of the APPROACHES intervention was defined as > 75% of residents living with dementia who had a documented ACP conversation, which was deemed a priori by the project team to be clinically significant.

### 1.6 Analytic Approach

Coincidence Analysis is a relatively new analytic approach within the larger family known as configurational comparative methods [31, 32]. Using Coincidence Analysis, researchers can identify the minimum set of necessary and sufficient conditions for an outcome to appear. The Coincidence Analysis algorithm removes any conditions that are redundant, superfluous, or extraneous, leaving only the key conditions known as “difference‐makers” [22, 33]. One notable feature of Coincidence Analysis is its ability to be applied to samples of different sizes, including small‐*n* studies [13, 19, 21, 22, 31]. Software used to support this analysis included the R packages “cna” (coincidence analysis) and “frscore” (fit robustness score) for Coincidence Analysis, RStudio, R, and Microsoft Excel [34, 35].

#### 1.6.1 Exploratory Data Analysis

We began our Coincidence Analysis by conducting an exploratory data analysis on the entire analytic dataset to help inform the selection of a subset of candidate factors related to implementation of the ACP Specialist Program for model development. Given our interest in the interplay between implementation processes and context, we focused on 48 of 64 intervention NHs that implemented at least one of two processes related to implementation of the ACP Specialist Program: site visits and/or monthly calls. Of these 48 sites, four had missing values for the outcome and were dropped, yielding an analytic dataset of 44 sites.

We applied the “minimally sufficient conditions” (i.e., “msc”) function within the R package “cna” to look across all 44 NHs and all 21 program‐related and context factors in the original dataset at once to identify bundles of specific conditions related to implementation of the ACP Specialist Program with especially strong connections to the outcome of > 75% of residents with a documented ACP conversation [15, 16, 21, 31]. This exhaustive “minimally sufficient conditions” routine evaluated every possible one‐, two‐, and three‐condition configuration present in the dataset, assessed each of them against a predetermined consistency threshold (i.e., how often presence of configuration was sufficient for presence of outcome), and then retained those configurations satisfying the consistency threshold at different consistency levels (95%, 90%, 85%, 80%, and 75%) in order to compare output at different thresholds [36]. This output allowed us to identify a small number of best‐of‐class configurations that met all of the following criteria to understand the conditions that best explained the outcome of > 75% of residents with a documented ACP conversation: top coverage score (i.e., explained the most cases with outcome present) within configurations of identical length (i.e., complexity level); presence of a sizable gap in coverage scores between the top‐scoring configuration and its next‐nearest neighbor within the same complexity level; alignment with logic, theory, and prior knowledge; and relevance to our research question. We then collected the factors present within these best‐of‐class configurations into a subset of candidate factors for model development and iteration.

#### 1.6.2 Model Development

Our goal was to develop an overall model that met all of the following criteria: ≥ 75% scores for both consistency and coverage; high fit‐robustness scores, indicating that the models were robust across multiple consistency and coverage thresholds; the same factors (taking on different values) explaining both the presence and absence of the outcome of > 75% of residents with a documented ACP conversation; and alignment with theory, background knowledge, case familiarity, and logic. This meant that our model needed to explain at least 75% of the cases with the outcome (coverage) and yield the outcome at least 75% of the time. These thresholds help to ensure the reliability (consistency) and explanatory breadth (coverage) of the final model.

We drew on the fit‐robustness score function within the R “frscore” package to assist with model selection. The fit‐robustness score conducts a reanalysis series by running the modeling functions within the R Coincidence Analysis package at varying intervals of consistency and coverage thresholds, compiling the top models generated at each threshold level and then assigning candidate models a normalized fit‐robustness score ranging between 0 and 1 based on how frequently individual models appear across different thresholds [35, 37, 38]. We varied the consistency and coverage thresholds between 70% and 95% using 1% increments, which yielded candidate models from 676 (i.e., 26 [2]) separate coincidence analysis runs, as there were 26 different possible thresholds between 70% and 95% for consistency and coverage, and each coincidence analysis run paired two of these prespecified thresholds (e.g., 70% consistency and 70% coverage, 70% consistency and 71% coverage, etc.). When reviewing the output, we looked for models with high normalized fit‐robustness scores, high consistency and coverage scores, and at least one mutable factor. We also prioritized models with relatively low complexity (i.e., number of conditions in a model) to avoid overfitting.

Data reduction and model development were conducted separately for the presence and absence of the outcome.

## 2 Results

A comparison of sites in the analytic sample with those that were not included showed that the two groups of sites were similar in terms of rurality, number of nurse practitioners in the building, number of beds, executive director turnover, staff turnover, and number of months participating in the intervention. Sites in the analytic sample were more likely than dropped sites to have robust implementation of the APPROACHES intervention (41% vs. 36%) but had higher ACP specialist turnover rates and lower ratings within the Centers for Medicare and Medicaid Services five‐star quality rating system for nursing homes (2.5 stars vs. 3.1 stars).

During the exploratory data analysis, four candidate factors stood out for their direct connection with the presence of the outcome of > 75% of residents with a documented ACP conversation and were selected for use in the model development phase: executive director turnover during the intervention period; participation in the monthly program calls; high hospitalization rate at baseline; and facility size (i.e., number of certified beds).

One of these factors was baseline hospitalization rate categorized by quartiles. It was evident from the output generated by the exploratory data analysis that NHs in the fourth quartile with the highest baseline hospitalization rate differed from NHs with lower baseline hospitalization rates. To reduce dimensionality, a binary metafactor was created in the modeling phase whereby if a NH was in the fourth quartile for baseline hospitalization rates, it was assigned a value of “1” and otherwise assigned a value of “0.”

For the absence of the outcome (i.e., < 75% of residents with a documented ACP conversation), the exploratory data analysis identified three candidate factors: executive director turnover during the intervention period; participation in the monthly program calls; and facility size (i.e., number of certified beds). Notably, these same three factors were among the factors appearing in the positive model, underscoring their role as difference‐makers as the outcome value changed when these factors took on different values.

### 2.1 Positive Model: Higher Proportion of ACP Conversations

**FIGURE 1:**
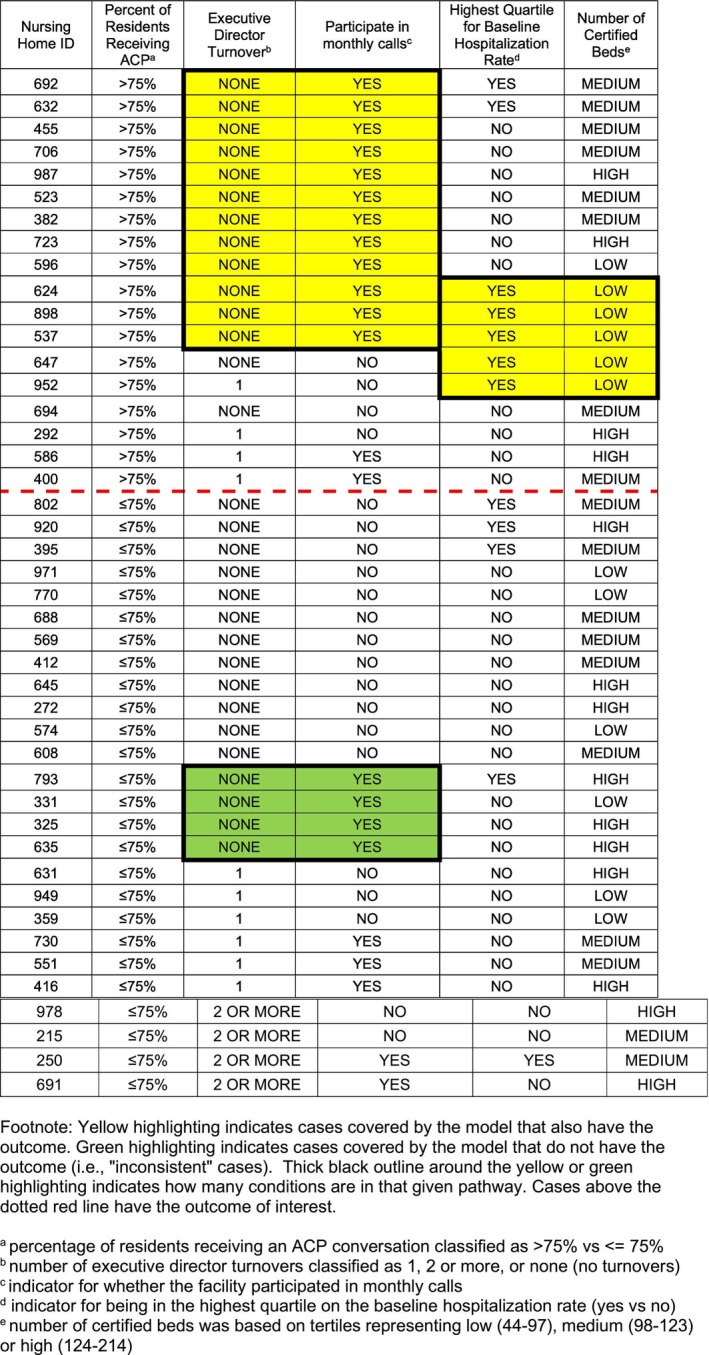
Solution visualization for positive model of difference‐makers associated with robust implementation of the advance care planning (ACP) specialist program for nursing home residents living with dementia

The Coincidence Analysis for the positive model yielded one solution that met all selection criteria, displayed in Figure 1. It was clearly the top model identified in the fit‐robustness score output, featuring the highest consistency and coverage scores along with the lowest complexity score (i.e., only four conditions total). For additional details about the positive model output, please see Text S1 and Table S1.

Original categorical values for each factor have been retained in Figure 1 for ease of reference. There were two distinct pathways leading to the presence of the outcome of > 75% of residents with a documented ACP conversation, with each pathway consisting of a bundle of two conditions:

- No executive director turnover during the intervention period and participation in the monthly program calls
  OR
- Having the highest hospitalization rate at baseline and having a small facility size (i.e., lowest number of certified beds).

Either one of these two pathways was sufficient for the outcome of > 75% of residents with a documented ACP conversation to appear. This model accounted for 14 of the 18 NHs with the outcome present, for an overall coverage score of 78% (14/18). It also met the prespecified consistency threshold, with 14 of 18 cases identified by the model having the outcome for an overall consistency score of 78% (14/18). As can be seen in Figure 1, the condition of “no executive director turnover” was not sufficient by itself for the outcome to appear; rather it had to appear jointly with “participation in the monthly calls.” The same was true for the other three conditions in the positive model: they distinguished between sites with and without the outcome only when paired together with other specific conditions.

### 2.2 Negative Model: Lower Proportion of ACP Conversations

**FIGURE 2:**
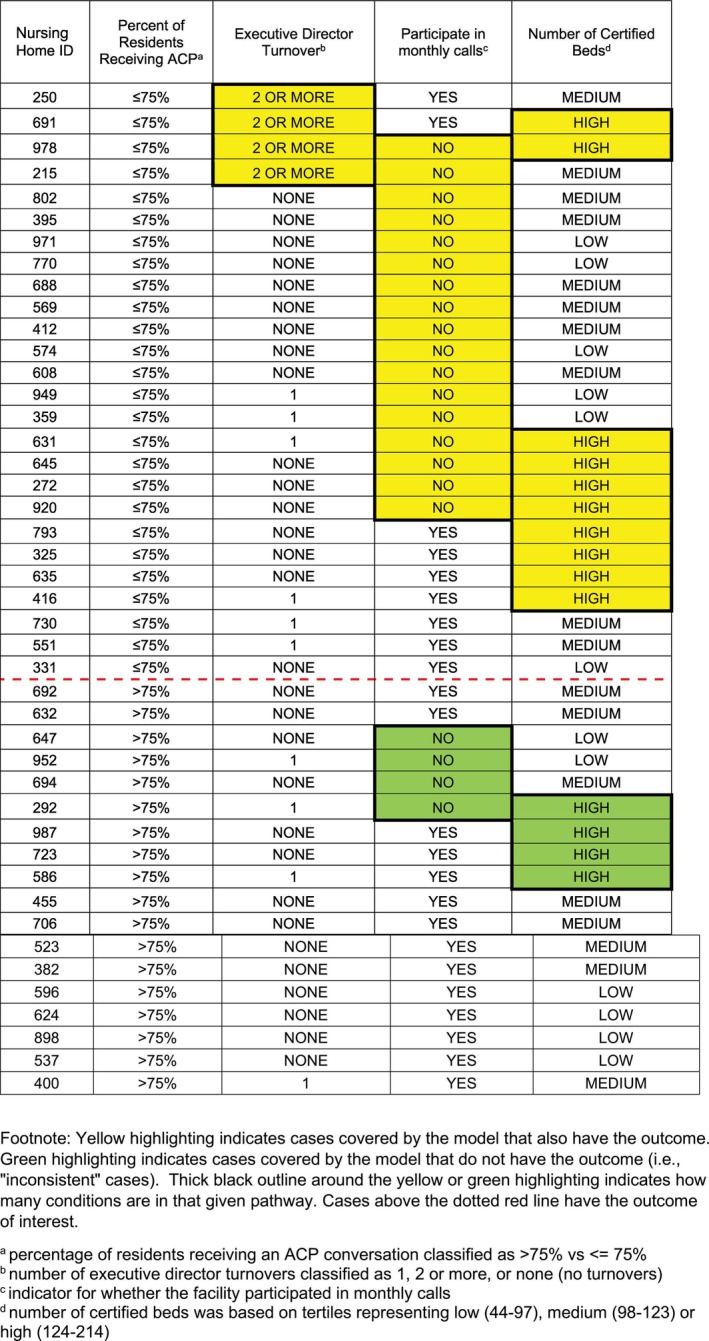
Solution Visualization for Negative model of difference‐makers associated with less robust implementation of the advance care planning specialist program for nursing home residents living with dementia.

The Coincidence Analysis for the negative model yielded one solution that met all selection criteria, displayed below in Figure 2. It was the third model listed in the fit‐robustness score output, combining the two top‐scoring models into a single solution with the highest coverage score among the top three models. For additional details about the negative model output, please see Text S2 and Table S2.

As before, original categorical values for each factor have been retained in Figure 2 for ease of reference. There were three distinct pathways leading to the absence of the outcome (i.e., < 75% with a documented ACP conversation), with each pathway consisting of a single condition:

- Two or more turnovers of the executive director position during the intervention period
  OR
- High number of certified beds

OR

- Non‐participation in the program monthly calls.

Any one of these three conditions was sufficient for the outcome of < 75% of residents with a documented ACP conversation to appear. This model accounted for 23 of the 26 NHs reporting < 75% with a documented ACP conversation, for an overall coverage score of 88% (23/26). It also met the prespecified consistency threshold with 23 of 30 cases identified by the model as having the outcome, for an overall consistency score of 77% (23/30). These same three factors (taking on different values) also appeared in the two pathways of the positive model.

## 3 Discussion

There is a pressing need for research in the NH setting to address inequities and optimize resident outcomes, but this setting is understudied and presents unique challenges to researchers [3]. Findings from this ePCT of an ACP NH intervention suggest that leadership stability and engagement with peers were essential drivers of robust pragmatic implementation of the ACP Specialist program as reflected by higher proportions of residents engaging in ACP conversations during the intervention period. Having greater improvement opportunities as reflected by higher rates of baseline hospitalization as well as a more manageable caseload for the ACP Specialist may also help explain successful implementation. In contrast, leadership instability as reflected by administrator turnover, a high number of certified beds, and a lack of participation in monthly calls with peers was associated with poorer performance. These findings suggest that additional support, coupled with leadership stability, may be particularly important when implementing interventions in NHs that may lack opportunities to connect with peers or are more closed to opportunities to learn from others.

These findings contribute to our understanding of important conditions for robust implementation of both pragmatic and more general research conducted in NHs. We previously used Coincidence Analysis in the Centers for Medicare and Medicaid Services (CMS) demonstration project OPTIMISTIC to assess the conditions associated with successful implementation of our multi‐component intervention in 19 NHs. Strong investment by senior leadership and an environment in which there were high baseline rates of hospitalization, providing ample opportunities to improve, resulted in great reductions in potentially avoidable hospitalizations [13]. However, we did not assess conditions associated with implementation because we were able to control the consistency of the OPTIMISTIC project implementation across sites. In an explanatory trial of a goals of care communication intervention, investigators used a multiple case study design with pattern‐matching logic to assess factors related to implementation effectiveness in 11 participating NHs based on staff surveys, finding that issues related to adequate management and financial support prior to implementation were associated with implementation effectiveness [39]. Conditions reflective of the importance of support were relevant, underscoring the importance of strong engagement from NH leadership. In an ePCT in which the study team has minimal control over the day‐to‐day study conduct, like the APPROACHES project, this is especially important. It is possible that reducing the complexity of intervention delivery could lessen the apparent importance of NH leadership engagement, particularly if known drivers of complexity like changes to workflow and additional training can be minimized [6]. The Coincidence Analysis methodology provides a useful approach to assessing conditions associated with the successful implementation of a pragmatic trial intervention through secondary data sources without necessitating primary data collection from participants.

In the present study, it is possible there were other relevant conditions that affected implementation at participating facilities such as ACP Specialist professional background and partnerships with advanced practice providers and/or physicians. These conditions are not reflected in this analysis because this information was not routinely tracked by the participating companies, and building a system to assess these conditions would have been burdensome and inconsistent with an ePCT. It is also likely that there is a relationship between some of the conditions that emerged from the Coincidence Analysis model, making causal inference difficult. For example, leadership stability may reflect a more highly functioning facility in which ACP Specialists were able to fully implement the program, including engaging with their peers. Additionally, a number of facilities were excluded from this analysis due to a lack of implementation processes and outcomes data. None the less, our findings suggest helpful strategies to enhance implementation and provide evidence to support best practices in future NH ePCTs.

## 4 Conclusions

In this ePCT, two combinations of factors made a difference in the rate of implementation of an ACP intervention: leadership stability and engagement with peers as well as greater improvement opportunities in combination with a lower number of certified beds. These findings have implications for partnering with NHs in both future ePCTs and clinical trials regarding partner selection as well as intervention design. They also highlight the value and importance of proactively tracking implementation factors guided by models such as the contextual framework for implementation when doing intervention work in NHs [25].

## Author Contributions

Study design: S.E.H., W.T., K.T.U. Data management and analysis: T.E.S., E.J.M. Data interpretation and preparation of manuscript: S.E.H., T.S., W.T., K.T.U., E.J.M.

## Funding

This work was supported by the National Institute on Aging (Grant number: R33AG057463, Clinicaltrials.gov number: NCT03323502). Research reported in this publication was supported by the National Institute On Aging of the National Institutes of Health under Award Number R33AG057463. The content is solely the responsibility of the authors and does not necessarily represent the official views of the National Institutes of Health.

## Conflicts of Interest

Kathleen Unroe is the founder of Probari, a healthcare start‐up focused on clinical support of nursing home care. The other authors declare no conflicts of interest.

## Supporting information


**Table S1:** Output details for positive models.
**Table S2:** Output details for negative models.
