# Supplementary material for: Difference‐Makers for Robust Implementation of a Nursing Home Advance Care Planning Embedded Pragmatic Clinical Trial
Source: J Am Geriatr Soc. 2026 Jan 30;74(3):712–21. doi: 10.1111/jgs.70289 (PMC12968356; doi:10.1111/jgs.70289)
Supplement: Supplementary file 1 — Table S1: Output details for positive models. Table S2: Output details for negative models. [file JGS-74-712-s001.pdf]

Supplementary Text S1: Output details for positive models

The frscore for the positive model (i.e., OUTCOME=1) yielded the 4 models below. The models are presented first, followed below by their consistency, coverage, complexity, and normalized frscore.

We selected the first model (highlighted) because it is clearly the top-scoring model in terms of its normalized frscore and met all of our selection criteria.

1
ED_TURNOVER=1*PARTICIPATE_MONTHLY_CALLS=1+HOSP_RATE_FOUR=1*NUMBER_BEDS=1<->OUTCOME=1

2
ED_TURNOVER=1*PARTICIPATE_MONTHLY_CALLS=1+HOSP_RATE_FOUR=1*NUMBER_BEDS=1+PARTICIPATE_MONTHLY_CALLS=1*HOSP_RATE_FOUR=0*NUMBER_BEDS=2<->OUTCOME=1

3
ED_TURNOVER=1*PARTICIPATE_MONTHLY_CALLS=1+ED_TURNOVER=2*HOSP_RATE_FOUR=1<->OUTCOME=1

4
PARTICIPATE_MONTHLY_CALLS=1*NUMBER_BEDS=2+PARTICIPATE_MONTHLY_CALLS=1*NUMBER_BEDS=1+HOSP_RATE_FOUR=1*NUMBER_BEDS=1<->OUTCOME=1

Supplementary Table S1: Output details for positive models

	Consistency (0-1.000)	Coverage (0-1.000)	Complexity (1-12)	Normalized frscore (0-1.00)
1	0.778	0.778	4	1.00
2	0.714	0.833	7	0.58
3	0.765	0.722	4	0.34
4	0.765	0.722	6	0.04

Supplementary Text S2: Output details for negative models

The frscore for the negative model (i.e., OUTCOME=0) yielded a total of 28 candidate models. All results are listed below. As before, the models are presented first, followed below by their consistency, coverage, complexity, and normalized frscore.

We selected the third model listed (highlighted) because it had a high normalized frscore (0.767), combined the results from the first two models listed and had the highest coverage score among the top three models. It met all of our selection criteria and also featured relatively low complexity (i.e., only 3 conditions total).

1
ED_TURNOVER=3+PARTICIPATE_MONTHLY_CALLS=2<->OUTCOME=0

2
PARTICIPATE_MONTHLY_CALLS=2+NUMBER_BEDS=3<->OUTCOME=0

3
ED_TURNOVER=3+PARTICIPATE_MONTHLY_CALLS=2+NUMBER_BEDS=3<->OUTCOME=0

4
ED_TURNOVER=3+PARTICIPATE_MONTHLY_CALLS=2+HOSP_RATE_FOUR=0*NUMBER_BEDS=1+HOSP_RATE_FOUR=1*NUMBER_BEDS=3<->OUTCOME=0

5
ED_TURNOVER=3+PARTICIPATE_MONTHLY_CALLS=2+HOSP_RATE_FOUR=0*NUMBER_BEDS=1<->OUTCOME=0

6
ED_TURNOVER=3+ED_TURNOVER=1*PARTICIPATE_MONTHLY_CALLS=2+HOSP_RATE_FOUR=0*NUMBER_BEDS=1+HOSP_RATE_FOUR=1*NUMBER_BEDS=3<->OUTCOME=0

7
ED_TURNOVER=3+PARTICIPATE_MONTHLY_CALLS=2+HOSP_RATE_FOUR=1*NUMBER_BEDS=3<->OUTCOME=0

8
ED_TURNOVER=3+PARTICIPATE_MONTHLY_CALLS=2*HOSP_RATE_FOUR=0+HOSP_RATE_FOUR=0*NUMBER_BEDS=1+HOSP_RATE_FOUR=1*NUMBER_BEDS=3<->OUTCOME=0

9
PARTICIPATE_MONTHLY_CALLS=2+NUMBER_BEDS=3+HOSP_RATE_FOUR=0*NUMBER_BEDS=1<->OUTCOME=0

10
ED_TURNOVER=3+PARTICIPATE_MONTHLY_CALLS=2+NUMBER_BEDS=3+HOSP_RATE_FOUR=0*NUMBER_BEDS=1<->OUTCOME=0

11
ED_TURNOVER=3+PARTICIPATE_MONTHLY_CALLS=2+ED_TURNOVER=1*NUMBER_BEDS=3+HOSP_RATE_FOUR=0*NUMBER_BEDS=1<->OUTCOME=0

12
PARTICIPATE_MONTHLY_CALLS=2+HOSP_RATE_FOUR=0*NUMBER_BEDS=1+HOSP_RATE_FOUR=1*NUMBER_BEDS=3<->OUTCOME=0

13
ED_TURNOVER=3+PARTICIPATE_MONTHLY_CALLS=2*HOSP_RATE_FOUR=0+PARTICIPATE_MONTHLY_CALLS=2*NUMBER_BEDS=2+HOSP_RATE_FOUR=1*NUMBER_BEDS=3<->OUTCOME=0

14
ED_TURNOVER=3+PARTICIPATE_MONTHLY_CALLS=2+ED_TURNOVER=1*NUMBER_BEDS=3<->OUTCOME=0

15
ED_TURNOVER=3+PARTICIPATE_MONTHLY_CALLS=2*HOSP_RATE_FOUR=0+PARTICIPATE_MONTHLY_CALLS=2*NUMBER_BEDS=2+ED_TURNOVER=1*PARTICIPATE_MONTHLY_CALLS=2*NUMBER_BEDS=3<->OUTCOME=0

16
ED_TURNOVER=3+PARTICIPATE_MONTHLY_CALLS=2*NUMBER_BEDS=2+PARTICIPATE_MONTHLY_CALLS=2*NUMBER_BEDS=3+HOSP_RATE_FOUR=0*NUMBER_BEDS=1<->OUTCOME=0

17
ED_TURNOVER=3+ED_TURNOVER=1*PARTICIPATE_MONTHLY_CALLS=2+PARTICIPATE_MONTHLY_CALLS=2*NUMBER_BEDS=3+HOSP_RATE_FOUR=0*NUMBER_BEDS=1<->OUTCOME=0

18
ED_TURNOVER=3+ED_TURNOVER=1*PARTICIPATE_MONTHLY_CALLS=2+PARTICIPATE_MONTHLY_CALLS=2*HOSP_RATE_FOUR=0+HOSP_RATE_FOUR=1*NUMBER_BEDS=3<->OUTCOME=0

19
PARTICIPATE_MONTHLY_CALLS=2+ED_TURNOVER=1*NUMBER_BEDS=3<->OUTCOME=0

20
ED_TURNOVER=3+ED_TURNOVER=1*PARTICIPATE_MONTHLY_CALLS=2+HOSP_RATE_FOUR=0*NUMBER_BEDS=1<->OUTCOME=0

21
ED_TURNOVER=3+ED_TURNOVER=1*PARTICIPATE_MONTHLY_CALLS=2+PARTICIPATE_MONTHLY_CALLS=2*HOSP_RATE_FOUR=0+HOSP_RATE_FOUR=0*NUMBER_BEDS=1<->OUTCOME=0

22
ED_TURNOVER=3+PARTICIPATE_MONTHLY_CALLS=2*HOSP_RATE_FOUR=0+PARTICIPATE_MONTHLY_CALLS=2*NUMBER_BEDS=2+HOSP_RATE_FOUR=0*NUMBER_BEDS=1<->OUTCOME=0

23
ED_TURNOVER=3+ED_TURNOVER=1*PARTICIPATE_MONTHLY_CALLS=2+PARTICIPATE_MONTHLY_CALLS=2*HOSP_RATE_FOUR=0<->OUTCOME=0

24
ED_TURNOVER=3+PARTICIPATE_MONTHLY_CALLS=2*HOSP_RATE_FOUR=0+PARTICIPATE_MONTHLY_CALLS=2*NUMBER_BEDS=2+PARTICIPATE_MONTHLY_CALLS=2*NUMBER_BEDS=3<->OUTCOME=0

25
PARTICIPATE_MONTHLY_CALLS=2+ED_TURNOVER=1*NUMBER_BEDS=3+HOSP_RATE_FOUR=0*NUMBER_BEDS=1<->OUTCOME=0

26
PARTICIPATE_MONTHLY_CALLS=2*HOSP_RATE_FOUR=0+PARTICIPATE_MONTHLY_CALLS=2*NUMBER_BEDS=2+HOSP_RATE_FOUR=0*NUMBER_BEDS=1+HOSP_RATE_FOUR=1*NUMBER_BEDS=3<->OUTCOME=0

27
PARTICIPATE_MONTHLY_CALLS=2*NUMBER_BEDS=2+PARTICIPATE_MONTHLY_CALLS=2*NUMBER_BEDS=3+HOSP_RATE_FOUR=0*NUMBER_BEDS=1+HOSP_RATE_FOUR=1*NUMBER_BEDS=3<->OUTCOME=0

28
ED_TURNOVER=1*PARTICIPATE_MONTHLY_CALLS=2+PARTICIPATE_MONTHLY_CALLS=2*HOSP_RATE_FOUR=0+HOSP_RATE_FOUR=0*NUMBER_BEDS=1+HOSP_RATE_FOUR=1*NUMBER_BEDS=3<->OUTCOME=0

Supplementary Table S2: Output details for negative models

	Consistency (0-1.000)	Coverage (0-1.000)	Complexity (1-12)	Normalized frscore (0-1.000)
1	0.826	0.731	2	1.000
2	0.759	0.846	2	0.987
3	0.767	0.885	3	0.767

4	0.808	0.808	6	0.636
5	0.800	0.769	4	0.608
6	0.870	0.769	7	0.591
7	0.833	0.769	4	0.580
8	0.864	0.731	7	0.569
9	0.742	0.885	4	0.554
10	0.750	0.923	5	0.547
11	0.767	0.885	6	0.511
12	0.792	0.731	5	0.455
13	0.909	0.769	7	0.442
14	0.786	0.846	4	0.422
15	0.905	0.731	8	0.405
16	0.870	0.769	7	0.369
17	0.833	0.769	7	0.360
18	0.870	0.769	7	0.338
19	0.769	0.769	3	0.336
20	0.864	0.731	5	0.319
21	0.833	0.769	7	0.287
22	0.864	0.731	7	0.248
23	0.864	0.731	5	0.246
24	0.905	0.731	7	0.241
25	0.750	0.808	5	0.241
26	0.864	0.731	8	0.233
27	0.864	0.731	8	0.198
28	0.826	0.731	8	0.181
